# Rescue surgery for advanced anal gland adenocarcinoma: A case report^☆^

**DOI:** 10.1016/j.ijscr.2019.03.019

**Published:** 2019-03-28

**Authors:** Sonaira Francisca Alves da Silva Bernardes, Dirceu de Castro Rezende Junior, Natasha Sa Gille Rissin, Tânia Rosa Pereira da Mota, Alexandre de Brito Borges Pimentel

**Affiliations:** Hospital das Forças Armadas, Estrada Contorno do Bosque s/nº, 70658900, Brasília, DF, Brazil

**Keywords:** Anal gland neoplasms, Neoadjuvant therapy, Surgical oncology, Case report

## Abstract

**Introduction:**

Extramucosal anal canal adenocarcinomas can arise in anorectal fistulas and the anal glands, the latter being rare. We present a rare case of anal gland adenocarcinoma treated with a combination of neoadjuvant therapy and radical surgical resection.

**Presentation of case:**

A 56-year-old man presented with rectal bleeding and irritation, and a nodule that had been enlarging for 10 months. Rectal examination revealed a bleeding ulceroproliferative growth at the left lateral edge of the anus without apparent invasion of the anal mucosa. Histopathology and immunohistochemistry confirmed the diagnosis of anal canal adenocarcinoma CK7+,CDX2−, and focalCK20+). Endoanal ultrasound showed a lesion involving the anal canal, extending into the transition zone with the lower rectum, invading the external anal sphincter, with no cleavage plane with the urethra, measuring 89 × 33 × 57 mm, associated with lymphadenopathy in the lower mesorectum (uT4N1). PET/CT confirmed a hypermetabolic lesion on the anal edge and bilateral hypermetabolic inguinal lymph nodes suggestive of secondary involvement. Colonoscopy was normal. The patient was started on neoadjuvant therapy with oral capecitabine and radiotherapy (57.6 Gy). Twelve weeks, the patient underwent extralevator abdominoperineal excision, cystoscopy (free urethral mucosa), skeletonization of the urethra with partial resection of the corpus cavernosum, and pelvic floor reconstruction with a vertical rectus abdominis myocutaneous flap.

**Discussion:**

Treatment of anal gland adenocarcinoma remains to be established. A combination of radical surgical resection and neoadjuvant/adjuvant chemoradiotherapy has been suggested, as performed here.

**Conclusion:**

Patients with advanced anal gland adenocarcinoma may benefit from neoadjuvant therapy followed by rescue surgery.

## Highlights

• Challenging diagnosis of advanced anal gland adenocarcinoma.
• Multidisciplinary surgical approach to treat advanced anal gland adenocarcinoma.
• Extralevator abdominoperineal excision (ELAPE) was possible.
• Vertical rectus abdominis myocutaneous flap reconstruction following ELAPE.

## 1 Introduction

Anal canal cancer accounts for 1–2% of all colon tumors. Approximately 75% of tumors are squamous cell carcinomas and 20% are adenocarcinomas [1]. Adenocarcinomas may be of colorectal type arising in the anal mucosa above the dentate line or extramucosal adenocarcinoma arising in the epithelium of the anal canal, including the mucosal surface, the anal glands, and the lining of fistulous tracts [2]. Extramucosal adenocarcinoma is a rare entity characterized primarily by extramucosal or intramural growth without a luminal component. The tumor infiltrates deeply beneath the submucosa and, because the overlying mucosa is not involved, can be misdiagnosed as a metastatic gastrointestinal carcinoma [3]. We present a rare case of anal gland adenocarcinoma successfully treated with a combination of neoadjuvant therapy and radical surgical resection in the coloproctology service at a tertiary care center supported by federal government funds with a wide range of specialty services. The work has been reported in line with the SCARE criteria [4].

## 2 Presentation of case

A 56-year-old male army veteran, obese (body mass index, 41.17 kg/m²) and hypertensive, presented to our clinic with the complaint of rectal bleeding and irritation, in addition to a nodule that had been progressively enlarging over the previous 10 months. Said patient had no history of abdominal or anorectal disorders other than the exposed, nor had he a family history of cancer. He denied smoking, but consumed alcohol weekly. Rectal examination revealed a bleeding ulceroproliferative growth at the left lateral edge of the anus without apparent invasion of the anal mucosa, measuring 10 × 8 cm. An incisional biopsy was performed, and histopathological examination revealed invasive tubular adenocarcinoma. On immunohistochemistry, the specimen stained positive for cytokeratin (CK) 7, negative for CDX2, and focally positive for CK20, confirming the diagnosis of adenocarcinoma of the anal canal.

Three-dimensional (3D) endoanal ultrasound showed a lesion involving almost the entire circumference of the anal canal, except for the right posterior quadrant, extending into the transition zone with the lower rectum, invading the external anal sphincter, with no cleavage plane with the urethra, measuring 89 × 33 × 57 mm, associated with lymphadenopathy in the lower mesorectum (uT4N1). Positron emission tomography/computed tomography (PET/CT) showed a hypermetabolic lesion on the anal edge with perineal extension compatible with primary neoplastic involvement and bilateral hypermetabolic inguinal lymph nodes suggestive of secondary involvement. Colonoscopy showed no changes in the mucosa of the colon and rectum, only the lesion on the anal edge was seen. During investigation, a severe aortic valve stenosis was also diagnosed and monitored pre- and postoperatively by the cardiology team.

The patient was started on neoadjuvant therapy with oral capecitabine and intensity-modulated radiation therapy to a total dose of 5760 cGy in 32 sessions (180 cGy/day) divided into 2 phases. The first phase consisted of 5040 cGy in 28 sessions involving lymphatic drainage of the pelvis and lesion area, and the second phase consisted of 720 cGy in 4 sessions delivered to the site of the primary lesion. The last radiation therapy session was not possible because of bleeding and repeated packed red blood cell transfusions.

On the 10-week restaging evaluation, 3D endoanal ultrasound showed an anal canal tumor extending into the anorectal transition zone and the base of the scrotum, with no cleavage plane with the urethra, AJCC stage yuT4N0. Twelve weeks after neoadjuvant therapy, the patient underwent a multidisciplinary surgical approach. The case was discussed among the teams and with the patient, who decided for penile preservation, except in case of gross invasion of the urethral mucosa (the patient was aware of the risks and oncological implications of this decision).

**Fig. 1:**
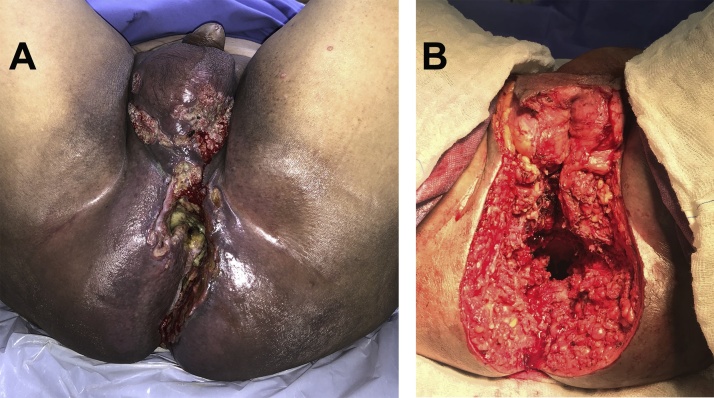
On the day of surgery: (A) appearance after radiation therapy and before resection and (B) appearance after tumor resection.

**Fig. 2:**
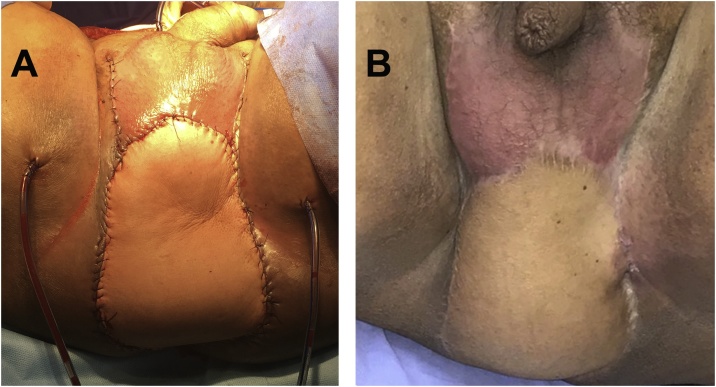
Appearance (A) immediately after pelvic floor reconstruction with a vertical rectus abdominis myocutaneous (VRAM) flap and (B) 5 months after surgery.

Surgical procedures were performed with the patient under epidural and general anesthesia. The patient was administered only Fleet enema for preparation and Unasyn for antibiotic prophylaxis. Extralevator abdominoperineal excision (ELAPE) was performed by the coloproctology team (Fig. 1). Urethrocystoscopy revealed no invasion of the urethral mucosa, and skeletonization of the urethra with partial resection of the corpus cavernosum was performed by the urology team. Pelvic floor reconstruction with a vertical rectus abdominis myocutaneous (VRAM) flap was then performed by the plastic surgery team (Fig. 2). All teams had extensive experience in the procedures performed. The patient recovered well without immediate or late postoperative complications. At hospital discharge, the patient was instructed by the plastic surgery team to refrain from sitting for 30 days; he was allowed to walk around and lie down.

The patient was followed at the outpatient clinic by close observation by the coloproctology, urology, plastic surgery, and oncology teams, including repeat PET/CT every 3 months. At 6 months, PET/CT showed nodules in the subcutaneous area of the graft and base of the scrotum, with uptake suggestive of neoplastic involvement. There were no clinical repercussions related to this condition, and the patient had a significant improvement in quality of life, resuming his work activities.

## 3 Discussion

The anal canal is histologically divided into 3 segments based on its lining epithelium: colorectal zone, lined by colorectal type glandular mucosa in the proximal portion; anal transition zone, lined by an epithelium with varying appearances in the middle portion; and a squamous epithelium-lined distal portion. For this reason, despite its short length, the anal canal can be affected by a variety of tumor types that reflect its complex anatomical and histological structure [2,5].

Anal gland adenocarcinoma is rare. A search of the literature between 1950 and 2011 conducted by Anwar et al. [6] yielded only 132 articles, most of which were case reports, 16 were retrospective observational studies, and none were randomized trials. Perhaps this is the reason why there is still no clear definition of diagnostic and treatment criteria. The histological profile commonly reveals an intramural adenocarcinoma with unaffected overlying anorectal mucosa [2,7]. Immunohistochemistry, as described here, shows positive staining for CK7 and negative staining for CK20 [8,9]. MUC5AC expression with CK5/6 has also been reported [9].

The treatment of anal gland adenocarcinoma remains to be established. However, Anwar et al. [6] after a review of the literature, concluded that the optimal treatment is a combination of radical surgical resection and neoadjuvant/adjuvant chemoradiotherapy - treatment which was performed in the case reported here, with a positive functional outcome despite local recurrence.

## 4 Conclusions

In conclusion, patients with advanced anal gland adenocarcinoma may benefit from neoadjuvant therapy followed by rescue surgery. Proper interaction between teams and good communication with the patient are essential to achieve satisfactory oncological, aesthetic, and functional outcomes in these cases.

## Conflicts of interest

The authors have no conflicts of interest to disclose.

## Sources of funding

The authors have no financial relationships relevant to this article to disclose.

## Ethical approval

Case reports are exempt from ethical approval in our institution (Armed Forces Hospital in Brasilia, Federal District, Brazil).

## Consent

Written informed consent was obtained from the patient for publication of this case report and accompanying images. The information and images presented do not allow identifying the patient.

## Registration of research studies

Not applicable.

## Guarantor

Sonaira Francisca Alves da Silva Bernardes.

Dirceu de Castro Rezende Junior.

Alexandre de Brito Borges Pimentel.

## Provenance and peer review

Not commissioned, externally peer-reviewed.
